# UNDERSTANDING AND SUPPORTING TECHNOLOGY EQUITY AND ACCESS FOR THE BENEFIT OF AGING ADULTS

**DOI:** 10.1093/geroni/igae098.2115

**Published:** 2024-12-31

**Authors:** Walter Boot, Xin Yao Lin, Jane Chung

**Affiliations:** Chair; Co-Chair; Discussant

## Abstract

Despite advancements, a significant “digital divide” persists, with older adults lagging in technology ownership, use, and proficiency compared to younger individuals. This session examines the digital divide’s causes, impacts, and solutions for aiding older adults. Melissa deCardi Hladek, PhD, CRNP, FNP-BC, proposes a personalized, values-driven intervention to enhance digital literacy among older adults, focusing on overcoming access and connectivity disparities, especially in communities of color, to foster inclusion and better health outcomes. Shannon Mejia, PhD, FGSA, investigates the emotional dynamics of older adults interacting with smart devices, such as voice assistants, and how these interactions are affected by other smart elements like fireplaces. Li Chu, PhD, highlights the importance of aligning technological information with age-relevant motivations, indicating that a focus on emotional meaning could boost older adults’ engagement with new technologies. Ladda Thiamwong, PhD, RN, FAAN, introduces the PEER intervention, a technology-based program aimed at preventing falls in low-income older adults through physio-feedback and peer-led exercises, emphasizing its role in transforming fall risk perceptions. Finally, Shenghao Zhang, PhD, questions the link between cognitive decline and the digital divide, suggesting that the relationship between age, general fluid abilities, and technology proficiency is more complex, offering new perspectives on technology learning and usage among older adults. Jane Chung, PhD, RN, will serve as discussant.

